# Complete Genome Sequence of Streptococcus agalactiae Serotype III, Multilocus Sequence Type 335 Strain HU-GS5823, Isolated from a Human Patient in Japan with Severe Invasive Infection

**DOI:** 10.1128/MRA.01303-18

**Published:** 2018-11-21

**Authors:** Kentaro Nagaoka, Satoshi Konno, Kazunori Murase, Taisei Kikuchi, Ichiro Nakagawa

**Affiliations:** a Department of Respiratory Medicine, Faculty of Medicine and Graduate School of Medicine, Hokkaido University, Sapporo, Hokkaido, Japan; b Department of Infectious Diseases, Faculty of Medicine, University of Miyazaki, Miyazaki, Japan; c Department of Microbiology, Graduate School of Medicine, Kyoto University, Kyoto, Japan; Loyola University Chicago

## Abstract

Streptococcus agalactiae is an important causal pathogen of neonatal and obstetric sepsis, and it may be involved in invasive infection in immunocompromised and elderly individuals. Here, we report the complete genome sequence of Streptococcus agalactiae serotype III strain HU-GS5823, which was isolated from a patient in Japan with an invasive infection.

## ANNOUNCEMENT

Streptococcus agalactiae (group B Streptococcus [GBS]), a commensal species in the gastrointestinal and genitourinary tracts of nearly 30% to 40% of healthy human adults, is also the most common cause of neonatal and obstetric sepsis (1). This changing epidemiology of invasive disease has highlighted the increasing incidence of infection caused by this pathogen in immunocompromised and elderly individuals (2, 3). It has also been documented to be important in veterinary medicine (4–6). Sequence type 17 (ST17) strains of S. agalactiae belong to a hypervirulent lineage of homogenous serotype III clones and are associated with a disproportionately high occurrence of invasive neonatal disease, particularly meningitis (6–8), but invasive disease in elderly individuals is yet to be studied. Here, we report the 2.2-Mb sequence of the complete circular chromosome of S. agalactiae strain HU-GS5823 with serotype III (ST335), produced by the combined technologies of Oxford Nanopore MinION and Illumina MiSeq.

In 2017, S. agalactiae strain HU-GS5823 was isolated from the blood of an adult patient with a severe invasive infection presenting as necrotizing fasciitis and infectious endocarditis. The strain HU-GS5823 was cultured for 8 h at 37°C in BD Bactec Plus aerobic medium (Becton, Dickinson and Company), and genomic DNA was extracted using the DNeasy blood and tissue kit, following the manufacturer’s instructions (Qiagen). Extracted DNA was subjected to short-read and long-read sequencing on MiSeq (Illumina) and MinION (Oxford Nanopore Technologies) platforms, respectively. An Illumina library was prepared using a Nextera DNA library prep kit, and paired-end reads were generated using a MiSeq reagent kit (v3-600). A MinION library was prepared from unsheared genomic DNA using a rapid barcoding kit (SQK-RBK001) and was sequenced with an R9 flow cell (FLO-MIN106). To remove adapter and low-quality sequences, the obtained Illumina data were preprocessed using Trimmomatic (v0.36) (9) and used for a hybrid assembly with MinION long reads using the Unicycler pipeline (v0.4.7b) with default parameters (10). The assembled genome sequence was then annotated using Prokka (v1.11) with default settings (11). Illumina and MinION sequencing generated 1,924,586 paired-end reads covering 1,158,600,772 sequenced bases and 143,972 reads covering 1,062,473,853 sequenced bases, respectively, with an average length of 7,379 bp and a longest read length of 72,295 bp. The hybrid assembly resulted in one circular contig of 2,231,314 bp, with a mean coverage of 995× and an overall GC content of 35.6%. This genome contained 2,238 genes with 2,136 coding sequences (CDSs) and 21 rRNA and 81 tRNA/transfer-messenger RNA (tmRNA) genes.

In Japan, half of serotype III isolates, belonging to ST17 of clonal complex 17 (CC17), represent known high-virulence strains; the remaining isolates belong to ST19, ST27, and ST335 of CC19 (12). In addition, a new sequence type with an *mef*(A) and/or *mef*(E) gene, ST335, has gradually increased in frequency. The complete genome sequence of this strain will provide useful information for clinical studies and basic research.

### Data availability.

The complete genome sequence of strain HU-GS5823 has been deposited in DDBJ/EMBL/GenBank under the accession number AP018935. The raw read data can be found in the DDBJ SRA under the accession number DRA007465.
